# Control of sodium and potassium homeostasis by renal distal convoluted tubules

**DOI:** 10.1590/1414-431X2023e12392

**Published:** 2023-02-10

**Authors:** E.A. Gallafassi, M.B. Bezerra, N.A. Rebouças

**Affiliations:** 1 Faculdade Israelita de Ciências da Saúde Albert Einstein, São Paulo, SP, Brasil

**Keywords:** Distal convoluted tubule (DCT), Na-Cl cotransporter (NCC), Potassium channels, WNK4, RAAS

## Abstract

Distal convoluted tubules (DCT), which contain the Na-Cl cotransporter (NCC) inhibited by thiazide diuretics, undergo complex modulation to preserve Na^+^ and K^+^ homeostasis. The lysine kinases 1 and 4 (WNK1 and WNK4), identified as hyperactive in the hereditary disease pseudohypoaldosteronism type 2, are responsible for activation of NCC and consequent hypokalemia and hypertension. WNK4, highly expressed in DCT, activates the SPAK/OSR1 kinases, which phosphorylate NCC and other regulatory proteins and transporters in the distal nephron. WNK4 works as a chloride sensor through a Cl^-^ binding site, which acts as an on/off switch at this kinase in response to changes of basolateral membrane electrical potential, the driving force of cellular Cl^-^ efflux. High intracellular Cl^-^ in hyperkalemia decreases NCC phosphorylation and low intracellular Cl^-^ in hypokalemia increases NCC phosphorylation and activity, which makes plasma K^+^ concentration a central modulator of NCC and of K^+^ secretion. The WNK4 phosphorylation by cSrc or SGK1, activated by angiotensin II or aldosterone, respectively, is another relevant mechanism of NCC, ENaC, and ROMK modulation in states such as volume reduction, hyperkalemia, and hypokalemia. Loss of NCC function induces upregulation of electroneutral NaCl reabsorption by type B intercalated cells through the combined activity of pendrin and NDCBE, as demonstrated in double knockout mice (KO) animal models, *Ncc/pendrin* or *Ncc/NDCBE*. The analysis of ks-*Nedd-4-2* KO animal models introduced the modulation of NEDD4-2 by intracellular Mg^2+^ activity as an important regulator of NCC, explaining the thiazide-induced persistent hypokalemia.

## Introduction

The distal portions of the nephron, including the distal convoluted tubule (DCT), the connecting tubule (CNT), and the cortical collecting duct (CCD), are essential to fine-tune the excretion of sodium, potassium, chloride, hydrogen, bicarbonate, calcium, and magnesium ions. The discovery that the lysine kinases (WNK) (1) and the ubiquitin ligases involved in their degradation (2) were the target of genetic mutations that result in clinical manifestations of type II pseudohypoaldosteronism, whose symptoms can be alleviated by inhibition of the sodium-chloride cotransporter (NCC), was a significant trigger for the interest in the participation of distal convoluted tubules in the preservation of electrolyte homeostasis. Analyses of animals with selective deletion of genes in cells of the distal segments of the nephron have contributed invaluably to the understanding of the involvement of different transport mechanisms and regulatory proteins in electrolyte homeostasis and acid-base balance.

In this review, we bring together the current knowledge about DCT: the different types of cells that constitute it, conditions in which the reabsorption in DCT is decreased or increased, the relevant role of serum potassium levels to determine the intracellular chloride activity and, thereby, the activity of the WNK kinases, the isolated and combined action of angiotensin II and aldosterone on the modulation of the WNK4 kinase, and the analysis of the consequences of kidney-specific gene knockouts on the distal nephron function. The acquired knowledge in these areas has allowed us to make a picture of the complex coordinated control of transporters in DCT, CNT, and CCD to maintain electrolyte homeostasis.

## Transport mechanisms in distal convoluted tubule cells

**Figure 1:**
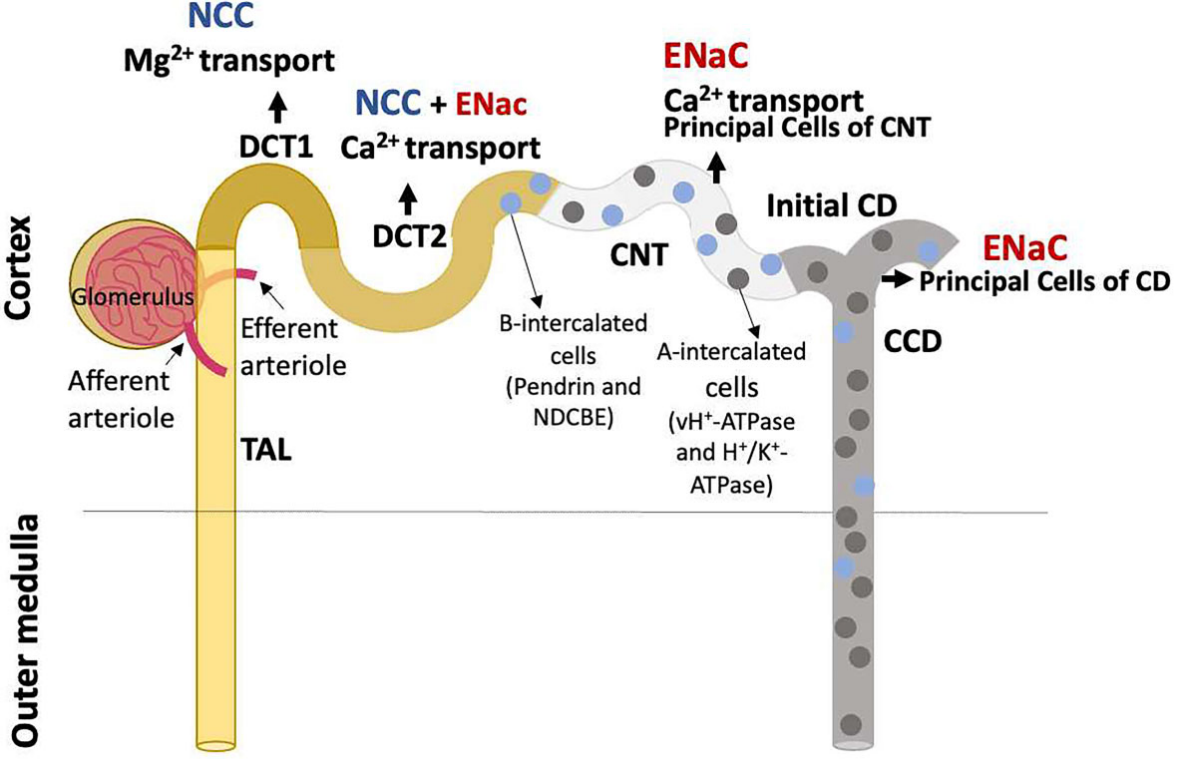
Anatomical relationships of distal convoluted tubules (DCT). DCTs follow the thick ascending limbs (TAL) of Henle and have cells that express the Na^+^-Cl^-^ cotransporter (NCC) in its initial length, and NCC and the epithelial Na^+^ channel (ENaC) in its final length. The complete nephron development depends on mutual interaction between the DCTs, derived from the metanephric mesenchyme, and connecting tubules and collecting ducts, derived from ureteric bud, where the water channel aquaporin 2 (AQP2), regulated by vasopressin, is expressed. DCTs are characterized by NCC expression. Intercalated cells type A and B, which together with principal cells (PC) form the mosaic of cells typical of connecting tubules (CNTs) and cortical collecting ducts (CCD), can be detected in DCT2. DCTs are important to Mg^2+^ and Ca^2+^ homeostasis, with Mg^2+^ reabsorbed mainly in DCT1 and Ca^2+^ in DCT2 and CNT. CD: collecting duct.

The DCT, the last portion of the nephron derived from the metanephric mesenchyme, is characterized by expressing NCC, which can be inhibited by thiazide diuretics, and is located immediately after the cortical portion of the thick ascending limb (TAL) of the loop of Henle, which expresses the Na^+^-K^+^-2Cl^-^ cotransporter (NKCC2). The TAL can end in the macula densa or extend a little further, but it does not reach the outer surface of the kidney. The CNT, which immediately follows the DCT and is derived from the ureteric bud, is accessible on the surface of the kidney, as well as at the end of DCT. Figure 1 depicts the relationship between DCT and the upstream and downstream tubular segments. The *in vivo* micropuncture technique does not allow the precise definition of the site being analyzed, which has given rise to some confusion regarding the transport mechanisms present in the DCT (3). The establishment of the *in vitro* tubular dissection and microperfusion, associated with analyses using the patch-clamp technique, in addition to the cellular localization of proteins with specific antibodies and the analysis of individual tubular cell transcriptomes allowed the more precise identification of these tubular segments.

**Figure 2:**
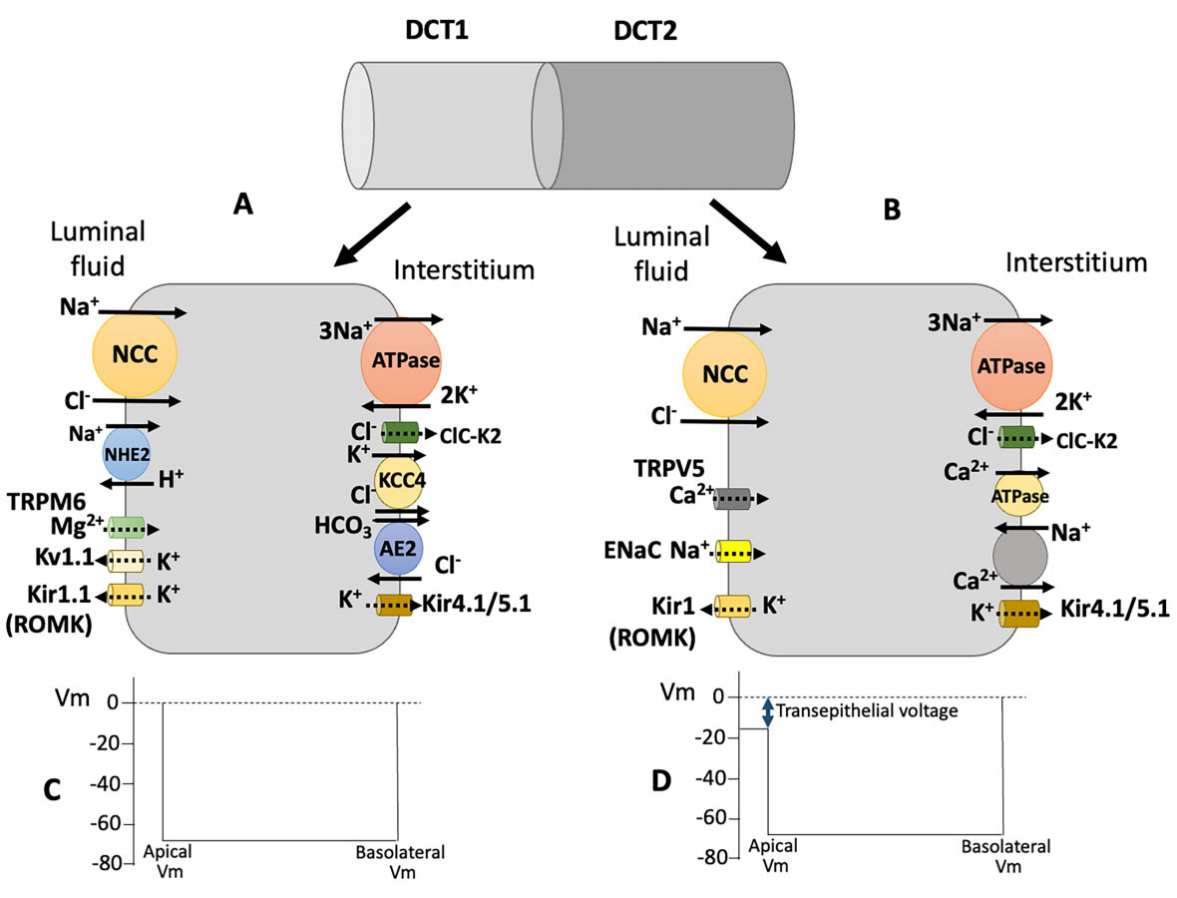
The main ion transporters in distal convoluted cells. **A**, Distal convoluted tubule (DCT1) cells express at the apical membrane the coupled Na^+^ transporters, Na-Cl cotransporter (NCC) and NHE2, the Mg^2+^ channel TRPM6 and the K^+^ channels Kir1.1 (ROMK), and Kv1.1; at the basolateral membrane, the Na/K-ATPase, the Cl^-^ channel ClC-K2, the K-Cl cotransporter KCC4, and the Cl^-^/HCO_3_^-^ exchanger AE2. The heterotetramer Kir4.1/Ki5.1 is the K^+^ channel in the basolateral membrane. **B**, DCT2 cells express at the apical membrane NCC and ENaC (epithelial Na^+^ channel), the Ca^2+^ channel TRPV5, and K^+^ channel Kir1.1; at the basolateral membrane, in addition to Na/K-ATPase and the K^+^ channel Kir4.1/Kir5.1, the active Ca^2+^ transporters, Na/Ca exchanger, and Ca-ATPase. **C**, The transepithelial electrical potential detected in DTC1 is usually zero. **D**, The transepithelial electrical potential detected in DCT2 is negative, due to the presence of ENaC, which depolarizes the apical membrane and favors K^+^ secretion.

The DCTs, in euvolemic conditions, receive around 8-10% of the filtered sodium and reabsorb 5-7%. DCT is identified as DCT1, in its initial 30-40%, and as DCT2, in its final 60-70%. The main pathway for transcellular Na^+^ reabsorption is the electroneutral NCC, present at the luminal membrane in all cells of DCT. The Na/H exchanger NHE2 is also present on the luminal membrane, especially in DCT1, and contributes, although less significantly, to electroneutral Na^+^ reabsorption in these segments (4). In DCT2 cells, the epithelial Na^+^ channel (ENaC), sensitive to the diuretic amiloride, is also present, despite that the influx of Na^+^ through NCC is still important (Figure 2).

In the basolateral membrane of distal tubule cells, Na^+^/K^+^-ATPase completes the Na^+^ reabsorption. The DCTs have the highest Na^+^/K^+^-ATPase activity per membrane area in the whole nephron (5), which is an evidence of the ability of the segment to accomplish higher NaCl reabsorption if it receives more Na^+^ from upstream segments. In the basolateral membrane, chloride transport is accomplished by a CLC type chloride channel, the CLC-Kb/2 (6), and by a K^+^-Cl^-^ cotransporter, the KCC4 (7). Considering that the transepithelial electrical potential difference in DCT1 is close to zero, both apical and basolateral membrane must have electrical potential close to K^+^ equilibrium potential, suggesting that in DCT1 cells, the Cl^-^ exit to the interstitium occurs mainly through the K^+^-Cl^-^ transporter, as suggested by Weinstein (8). The HCO_3_^-^ generated in the tubular cells by dissociation of carbonic acid, generated from CO_2_ hydration catalyzed by carbonic anhydrase II, is reabsorbed by the electroneutral HCO_3_^-^/Cl^-^ exchanger AE2 (9).

Relative to K^+^ handling in DCT, the K^+^-channel Kir1.1, also called renal outer medullary K channel 1 (ROMK1), is present in the apical membrane in both DCT1 and DCT2, but the increase in the amount of ROMK in the apical membrane in response to high K^+^ diet occurs only in DCT2 (10). The K^+^-channel Kv1.1, a voltage-sensitive K^+^ channel encoded by the *Kcna1* gene, was also identified in the apical membrane, mainly in DCT1, in association with the Mg^2+^-channel TRPM6 (11). ROMK1 and Kv1.1 determine the conductance and, therefore, the electrical potential difference of the apical membrane of DCT1. In DCT2 cells, in addition to ROMK, ENaC also contributes to the electrical potential difference across the apical membrane, depolarizing it and contributing to the negative transepithelial electrical potential difference, the driving force for K^+^ secretion observed in the final portion of DCT. The heterotetramer Kir4.1/Kir5.1 forms the K^+^ channel responsible for potassium conductance of the basolateral membrane of DCT1 and DCT2 cells; Kir4.1 forms the channel and Kir5.1 functions as a regulatory subunit (12).

The transcellular reabsorption of Mg^2+^ and Ca^2+^ in DCT is very important for the homeostasis of these divalent cations. The Mg^2+^ reabsorption in DCT, which occurs mainly in DCT1, depends on the transient receptor potential melastatin members 6/7 (TRPM6/7), especially TRPM6, a Mg^2+^ channel expressed at the luminal membrane of DCT cells (13); the basolateral mechanisms of Mg^2+^ transport are not defined. The transcellular Ca^2+^ reabsorption occurs mainly in DCT2 by the transient receptor potential vanilloid 5 (TRPV5) Ca^2+^ channel. Ca^2+^ is extruded across the basolateral membrane by the Na^+^/Ca^2+^ exchanger (NCX1), and the plasma membrane Ca^2+^-ATPases, PMCA1, and PMC4 (14); these Ca^2+^ transporters are present in the CNT, which is also relevant for transcellular Ca^2+^ reabsorption (15). In both segments, the expression of the cytoplasmic Ca^2+^ binding proteins, parvalbumin (DCT1) and calbindin D28K (DCT2 and CNT), are necessary for Ca^2+^ shuttling to the basolateral membrane (13). Figure 2 illustrates the mechanisms of Na^+^, K^+^, Cl^-^, HCO_3_^-^, Ca^2+^, and Mg^2+^ transport observed in DCT and the estimated transepithelial electrical potential difference.

The permeability of the paracellular pathway along the renal tubules depends on the proteins that form tight junctions, especially claudins. In the DCT, CNT, and CCD, claudins 3, 4, 7, 8, and 10 were identified (16). The role of each of these proteins in determining the selectivity and permeability of the paracellular pathway in the DCT is not defined. In CD, the claudins 4/8 seem to form a chloride channel in the paracellular pathway (16). When Na^+^ is reabsorbed via ENaC, it is expected that some Cl^-^ reabsorption occurs through the paracellular pathway in DCT2.

**Figure 3:**
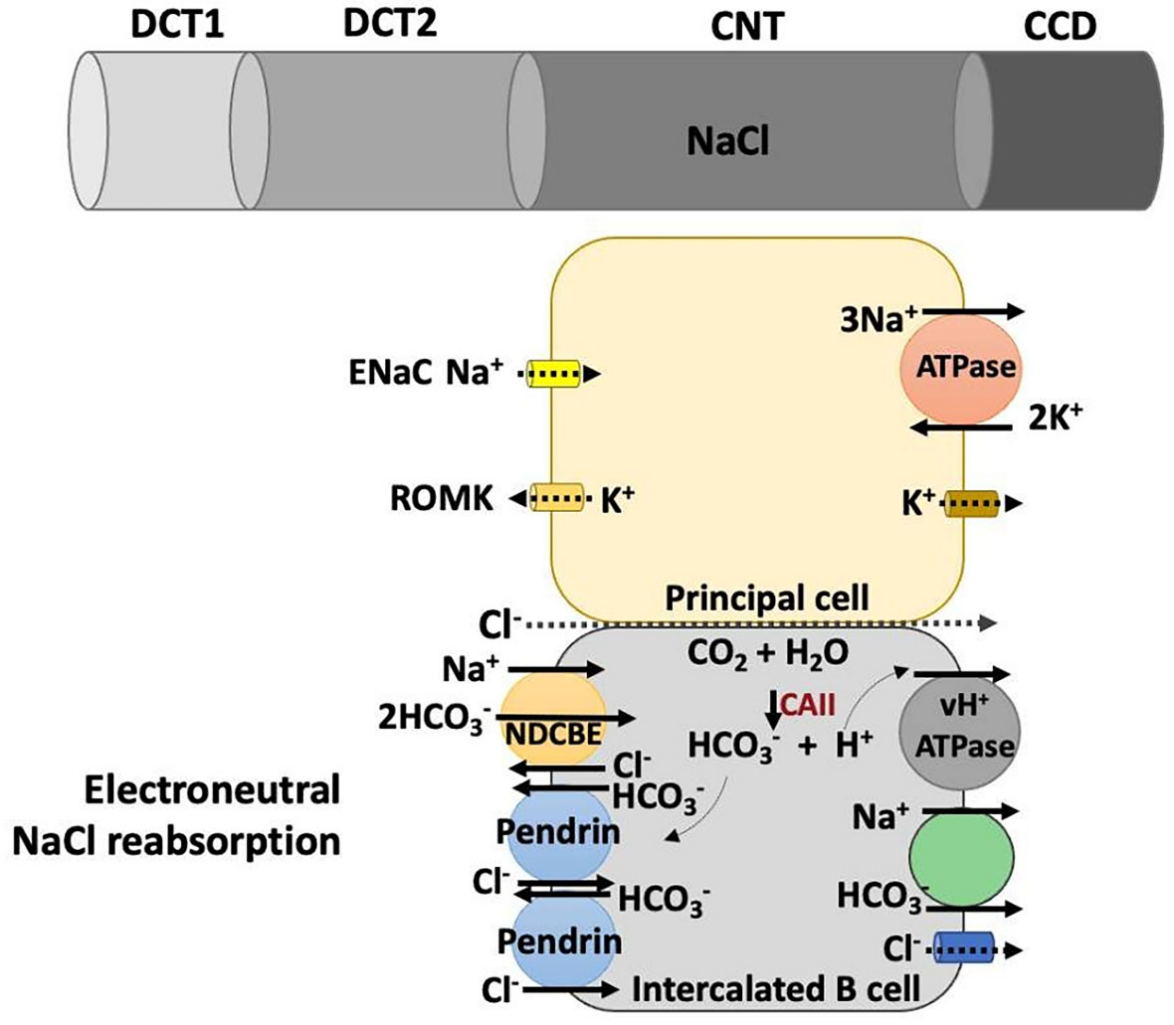
The main ion transporters in principal cells and type B intercalated cells (B-IC) in the connecting duct. ENaC and ROMK are present at the luminal membrane of principal cells, and Na^+^/K^+^-ATPase, at the basolateral membrane. In B-IC, the primary active transporter is the v-H^+^-ATPase present at the basolateral membrane. At the apical membrane, pendrin promotes HCO_3_^-^ secretion and Cl^-^ reabsorption, and NDCBE promotes reabsorption of 2 HCO_3_^-^ and 1 Na^+^, and secretion of 1 Cl^-^. The combined activity of 2 pendrin and 1 NDCBE results in electroneutral reabsorption of NaCl and nullifies the net HCO_3_^-^ transport at the luminal membrane. Chloride reabsorption also occurs by paracellular pathway, through claudins 4 and 8, which interact and form an anion selective channel. DCT: distal convoluted tubule; CNT: connecting tubule; CCD: cortical collecting tubule.

The final portion of DCT, at least in some species, also has intercalated cells randomly distributed among typical DCT cells (17). The connecting tubule and the cortical/outer medullary collecting duct are the renal tubular segments formed by a mosaic of different types of cells, the principal cells (PC) and the intercalated cells (ICs): type A intercalated cells (A-IC) secrete H^+^ and reabsorb HCO_3_^-^ by vacuolar-H^+^-ATPase present in the apical membrane and the HCO_3_^-^/Cl^-^ exchanger (AE1) in the basolateral membrane, respectively; type B intercalated cells (B-IC) secrete HCO_3_^-^ and reabsorb H^+^ (B-IC) by the HCO_3_^-^/Cl^-^ exchanger pendrin present at the apical membrane and the v-H^+^ATPase present in the basolateral membrane, respectively; non-A/non-B type ICs have v-ATPase and pendrin at the apical membrane, and can secret predominantly H^+^ or HCO_3_^-^, depending on the physiological conditions. Non-A/non-B ICs are predominant in CNT (18); however, it is not clear which type of ICs are present in DCT2, but B-ICs might be a pathway for Cl^-^ reabsorption in this segment, since it is important for transcellular Cl^-^ reabsorption in the downstream segments, CNT and CCD (Figure 3).

The apical membrane of DCT1 and DCT2 cells does not express aquaporins (AQP); therefore, these segments are constitutively water impermeable. The transcellular transport of Na^+^ and Cl^-^ in DCT always results in dilution of the tubular fluid, which makes it a diluting segment. The water permeability of the CNT and CD tubular segments depends on the presence of the antidiuretic hormone that makes the apical membrane of the principal cells water permeable through the insertion of type 2 aquaporins (AQP2). Intercalated cells do not express AQP2, so they are constitutively impermeable to water.

## Inhibition or loss of function of NCC

NCC is inhibited by thiazide diuretics (hydrochlorothiazide, chlorthalidone, and indapamide), the most widely prescribed class of diuretics for the control of arterial hypertension. The *Ncc* gene (Slc12a3) is the target of mutations in Gitelman syndrome (19), a disease with late clinical manifestations and of varying intensity, which is characterized by hydroelectrolytic changes, such as hypokalemia, metabolic alkalosis, hypocalciuria, hypomagnesemia, and reduced extracellular volume with arterial hypotension. The chronic use of hydrochlorothiazide (HCTZ) also results in similar hydroelectrolytic changes, in addition to the predisposition to hyponatremia, since this diuretic alters the kidney ability to dilute urine but does not alter the ability to concentrate it. The vasopressin secretion is stimulated by the extracellular volume contraction induced by NCC inhibition, so if there is free water intake, water reabsorption in the medullary collecting duct occurs even with reduced plasma osmolarity. In addition, César et al. (20) have shown that HCTZ increases the osmotic and diffusional water permeability in the inner medullary collecting duct, independently of ADH, in control and in Brattleboro rats, contributing to water preservation.

Given that mutations in the *Ncc* gene were identified as the cause of Gitelman syndrome (19), it was expected that the knockout (KO) of this gene (*Ncc*-KO) would cause the changes observed in patients with this syndrome, namely, decreased extracellular volume with mild arterial hypotension, hypokalemia, metabolic alkalosis, hypocalciuria, and hypomagnesemia. Mice with a deletion of this gene did not show hypokalemia and metabolic alkalosis, and arterial hypotension was observed only after 2 weeks on a sodium-depleted diet (21,22). The cells of the distal convoluted tubule in *Ncc*-KO animals were shown to be reduced in number and size and the mitochondria were smaller and less associated with basolateral membrane invaginations. The slight change in fluid homeostasis observed in *Ncc*-KO animals suggests compensatory mechanisms in the other tubular segments. Plasma levels of renin were shown to be elevated, which is evidence of a decrease in extracellular volume. These animals showed hypocalciuria and significant hypomagnesemia.

Magnesium depletion is always observed on inhibition, mutation, or deletion of the NCC, which highlights the fundamental role of DCT for Mg^2+^ homeostasis. In *Ncc*-KO mice, the described changes in DCT cells have been suggested as a possible cause of lower Mg^2+^ reabsorption (21); although hypotrophy of DCT has been observed in animals with *Ncc* deletion, this was not a consistent observation with the use of HCTZ (23). With calcium, the opposite, hypocalciuria, is usually observed when there is inhibition, deletion, or inactivation of NCC. It was clearly observed that reduced NCC activity leads to hypocalciuria even when the calcium channel of the apical membrane of the DCT (TRPV5) is deleted, and therefore the reduction in urinary Ca^2+^ excretion is not due to the higher transcellular Ca^2+^ reabsorption in the distal tubules after inhibition of NCC (23). TRPV5, as well as the other proteins involved with Ca^2+^ transcellular reabsorption, Na^+^/Ca^2+^ exchanger and calbindin, are not increased in animals treated with HCTZ (24). Hypocalciuria in these conditions is attributed to greater passive Ca^2+^ reabsorption in proximal tubules, stimulated by the reduction in extracellular volume due to the transient negative Na^+^ balance, which results in greater fractional reabsorption of Na^+^ and H_2_O in the proximal tubules. Since most of the filtered Ca^2+^ is passively reabsorbed in proximal tubules, in the hypovolemic state very little Ca^2+^ reaches the distal tubules. The largest fraction of filtered Mg^2+^, on the other hand, is reabsorbed in the TAL of the Henle's loop and the transport activity in this segment does not seem to be significantly stimulated when there is inhibition or inactivation of the NCC; therefore, there is no compensation that could recover the Mg^2+^ balance.

## NaCl reabsorption in CNT and CCD after NCC inhibition or deletion

The compensatory changes in tubular transport following NCC inhibition occurs in the downstream segments to the DCT and are mainly due to increased load of Na^+^ and Cl^-^ in these tubular segments. There is a transient increase in the fractional excretion of Na^+^ and Cl^-^, which eventually ends in contraction of the extracellular volume and subsequent changes in upstream tubular segments because of activation of extracellular volume regulatory mechanisms. Fractional excretion of Na^+^ (FE-Na) is recovered, but not always fractional excretion of K^+^ (FE-K), due to the greater load of Na^+^ in CNT and CCD, even after normalization of FE-Na.

The CNT and the CCD receive the additional Na^+^ load that is not reabsorbed by DCT. One of the most obvious consequence of a greater supply of Na^+^ to the principal cells of the CNT and CCD is increased electrogenic Na^+^ reabsorption by ENaC, which promotes more depolarization of the apical membrane of these cells and increased driving force for K^+^ secretion through ROMK channels; the same may occur in DCT2. Therefore, an important consequence of inhibition or mutation of NCC is the increased K^+^ secretion and hypokalemia. The increased Na^+^ reabsorption by ENaC contributes to restoring Na^+^ balance, but it disturbs the K^+^ balance. It has been well demonstrated that amiloride-induced natriuresis is increased when NCC is inhibited or deleted (25).

The B-ICs, predominant in the CNT (18), also contribute to restoring Na^+^ and Cl^-^ balance, through the reabsorption of NaCl through the combined action of pendrin and Na^+^-dependent Cl^-^/HCO_3_^-^ exchanger, NDCBE (26). NDCBE is inhibited by thyazides in higher doses than those required for NCC inhibition (27). The Na^+^, Cl^-^, HCO_3_^-^, and H^+^ transporters present in principal and B-IC cells are illustrated in Figure 3.

The relevant contribution of B-ICs for preservation of Na^+^ and K^+^ homeostasis when NCC is deleted was revealed in mice with NCC and pendrin double knockout (22). These mice present severe volume depletion, significant increase in renin-mRNA, low urine osmolarity secondary to defective insertion of AQP2 in PCs, without hypokalemia. Principal cells in pendrin KO mice present low ENaC activity, which is closely related with the loss of HCO_3_^-^ secretion and Cl^-^ reabsorption by the B-ICs. ENaC inhibition has been attributed to reduced luminal pH and to paracrine effects of ATP and prostaglandin E2 release, triggered by pendrin mutation in B-ICs (28-31). The reduced water permeability of principal cells might also be related with increased secretion of PGE2, which can be a consequence of the activation of the purinergic receptor PYP2 by ATP secreted by B-IC due to low activity of v-H^+^-ATPase (32,33).

The pendrin/NCC double KO mice analysis reveals the important role of the increased electroneutral NaCl reabsorption in B-IC to preserve K^+^ upon NCC inhibition. A phenotype similar to that observed with pendrin/NCC double KO is observed in mice with carbonic anhydrase II and NCC double knockout (*CA*II/*Ncc* dKO), since deletion of CAII abrogates pendrin function and the function of all transporters dependent on H^+^ and HCO_3_^-^ resultant from carbonic acid dissociation (34). The absence of hypokalemia is explained by the low ENaC activity upon deletion of pendrin. Double knockout of *Ncc* and *NDCBE*, on the other side, induces a mild increase in Na^+^ and Cl^-^ excretion relative to *Ncc*-KO, but severe hypokalemia (27), suggesting reabsorption of Na^+^ by principal cells, and Cl^-^ reabsorption by B-IC are efficient in preserving NaCl in Ncc-NDCBE-KO models, but the increased electrogenic Na^+^ reabsorption by ENaC, associated to electroneutral Cl^-^ reabsorption by pendrin, results in severe K^+^ loss due to increased driving for K+ secretion. These KO models show that the combined normal activity of pendrin/NDCBE in B-ICs is essential for electroneutral Na^+^ and Cl^-^ reabsorption and K^+^ preservation in volume depletion.

Phenotypes observed in double KO of NCC and pendrin have led to the suggestion of pharmacologic inhibition of pendrin and NCC to provide a novel and strong diuretic regimen for patients with fluid overload, including those with congestive heart failure, nephrotic syndrome, or renal failure (35), which would induce huge Na^+^ and water excretion without significant K^+^ loss. It is yet to be demonstrated that the pharmacological inhibition of pendrin can trigger the inhibition of ENaC and water permeability in principal cells as does the pendrin KO.

**Figure 4:**
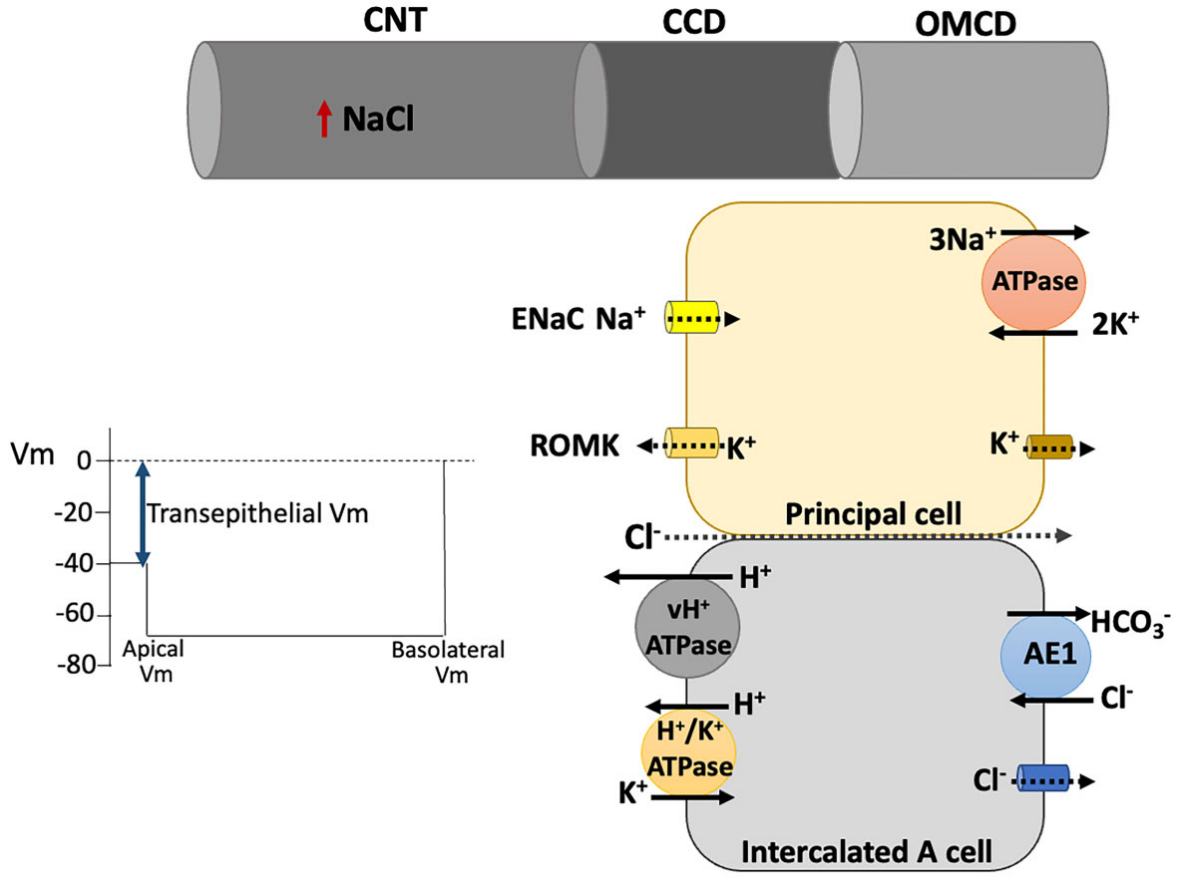
Inhibition of Na-Cl cotransporter (NCC) increases the driving force for H^+^ secretion by type A intercalated cells. The electrogenic H^+^ secretion by vH-ATPase at the apical membrane of type A intercalated cells (A-IC) is intensified by the higher Na^+^ reabsorption by epithelial Na^+^ channel (ENaC), due to increase in luminal negativity. H^+^ secretion and K^+^ reabsorption by H^+^/K^+^-ATPase is also stimulated by the hypokalemia induced by NCC inactivation.

Inhibition, mutation, or deletion of NCC can result in changes in acid-base status of the organism, and these changes are attributed to A-ICs, which are more abundant in CCD and the outer medullary collecting duct. The v-H^+^-ATPase activity of A-IC is facilitated by the increased luminal negativity in CCD (17,36) due to higher Na^+^ reabsorption by ENaC, and consequently, there is an increase in HCO_3_^-^ regeneration, which contributes to the metabolic alkalosis observed during chronic use of NCC inhibitors or in Gitelman syndrome (Figure 4). The hypokalemia associated with NCC inhibition also stimulates H^+^/K^+^-ATPase and contributes to H^+^ secretion and HCO_3_^-^ regeneration by the A-IC (37). In proximal tubules (PT), the hypokalemic reduction in ammonia recycling secondary to reduced activity of the enzyme glutamine synthetase, with subsequent increase in net ammonia secretion (38) and *de novo* HCO_3_^-^ generation, also contributes to maintenance of metabolic alkalosis. Alkalosis is sustained when volume contraction is established after NCC inhibition, because the sympathetic stimulation and higher levels of angiotensin II increases the fluid reabsorption in the PT, sustaining high Na^+^, HCO_3_^-^, and Cl^-^ absorption and reducing Cl^-^ load in the distal nephron, which reduces the pendrin ability to compensate the alkalotic disturbance.

## Hyperactivity of NCC and the activity of with-no-lysine kinases (WNKs)

Genetic hyperactivity of NCC is a rare condition seen in familial hyperkalemic hypertension (FHHt), also called type II pseudohypoaldosteronism (PHAII) or Gordon syndrome, characterized not only by hyperkalemia and metabolic acidosis, which are observed in the hypoaldosteronism, but also by hypertension (39). The involvement of WNKs in regulation of NCC was identified when it was discovered that the genetic mutations associated with this syndrome are located in the genes that encode kinases of the WNK family (1) or in the genes that encode proteins involved in ubiquitination for further degradation of these WNKs, Kelch-like 3 (KLHL3) and Cullin 3 (CUL3) (2,40). These mutations result in hyperexpression/hyperactivity of WNKs 1 and 4 and hyperactivation of NCC.

The kinases WNK1, KS-WNK1 (kidney specific WNK1), WNK2, WNK3, WNK4, with the exception of WNK2, have been identified in the kidney and they control the transport activity in the distal nephron (41). KS-WNK1, a WNK1 amputated from N-terminal amino acids, where the kinase activity is located, results from an alternative promoter of the *Wnk1* gene and acts as an inhibitor of WNK1 and is restricted to DCT (42). WNK1 and WNK4 activate the SPAK (Ste20-related proline-alanine-rich kinase) and OSR1 (oxidative stress response kinase-1) kinases which, in turn, phosphorylate transporters present in DCT, CNT, and CCD, modulating the reabsorption of Na^+^ and K^+^ secretion in the distal portions of the nephron.

Despite various controversies in the literature regarding the action of WNK4 on NCC (41), the most recent data converge to the understanding that WNK4 is actually the WNK kinase most significantly involved in the regulation of NCC. WNK4 exerts both inhibitory and stimulatory effects on NCC. WNK4 inhibits NCC by reducing its insertion in the apical membrane by diverting it to lysosomes (43), and, most important, it activates NCC by activating SPAK and controlling NCCs’ level of phosphorylation and, thereby, the activity of this cotransporter (44).

**Figure 5:**
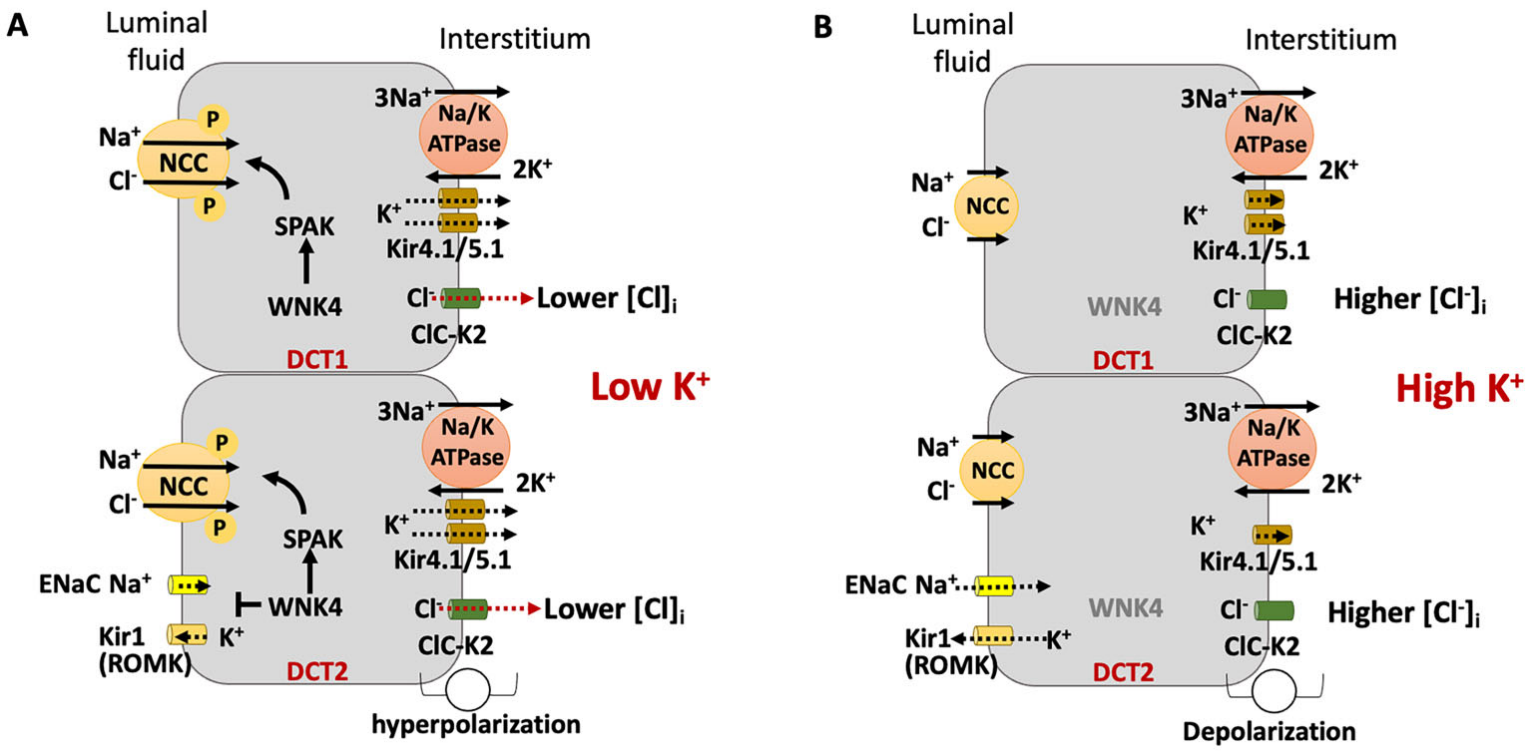
WNK4 activity is modulated by intracellular Cl^-^ activity. **A**, Low plasma K^+^ hyperpolarizes the basolateral membrane of distal convoluted tubules (DCT) cells and decreases intracellular Cl^-^, thereby activating WNK4/SPAK, which phosphorylates and activates Na-Cl cotransporter (NCC). Activated WNK4 induces proteasomal degradation of ENaC and ROMK. **B**, High plasma K^+^ depolarizes the basolateral of DCT cells and increases intracellular Cl^-^, thereby abolishing the NCC activation by WNK4/SPAK, and decreasing proteasomal degradation of ENaC and ROMK induced by WNK4.

An attempt to unify the hypothesis of WNKs action in DCT cells came from the observation that WNKs have a Cl^-^ interaction domain, which makes them sensitive to the intracellular levels of this ion and a proper intracellular chloride sensor (45). The elevation of [Cl^-^]_i_, by inhibiting the autophosphorylation of these kinases, results in their inhibition (46). The intracellular levels of Cl^-^, in turn, are directly affected by the plasma K^+^ levels, since the intracellular Cl^-^ concentration is determined by the basolateral transmembrane electrical potential difference: decrease in serum K^+^ (Figure 5A) results in hyperpolarization of the basolateral membrane and decreased [Cl^-^]_i_; increase in serum K^+^ (Figure 5B) results in depolarization of the basolateral membrane and increase in [Cl^-^]_i_ (47,48). Studies developed in the last decade have made clear the relevance of K^+^ load for rapid NCC regulation (49).

WNK4 shows high levels of expression in DCT (50) and presents sensitivity to [Cl^-^]_i_ in the concentration range observed in the cells of the distal nephron, between 10-20 mM, in contrast to WNK1, which is inhibited by Cl^-^ in higher concentrations (47,51). This characteristic makes WNK4 the ideal sensor for serum K^+^ concentration via changes in [Cl^-^]_i_. The effects of [Cl^-^]_i_ are translated by SPAK, which phosphorylates NCC (52). It is not excluded that intracellular K^+^ activity itself might have influence on WNK activity, by an effect on the melting point of the kinase domain of WNKs, as experimentally observed in mammalian and Drosophila WNKs, with greatest sensitivity of WNK4 in range of 80-180 mM of K^+^ (53).

A specific feature of the WNK-SPAK/OSR1 signaling pathway in the cells of the distal tubules is that its components can be concentrated in spherical cytoplasmic domains not bounded by a membrane, recently called “WNK bodies”. They have KS-WNK1 as an essential component, and WNK4 appears to be the active form of kinase primarily responsible for SPAK/OSR1 activation (54,55). The formation of WNK bodies with phosphorylated SPAK, observed in DCT cells, is stimulated by K^+^ depletion and is promptly suppressed by K^+^ overload. The interaction between WNK4 and KS-WNK1, the latter with practically exclusive expression in DCT cells, was highlighted in observations made in oocytes, in which the injection of KS-WNK1 leads to significant activation of SPAK and NCC, by activation of WNK4 (56). KS-WNK1 activates WNK4 by inducing phosphorylation of Ser^335^; this same residue becomes phosphorylated when the intracellular concentration of Cl^-^ is reduced. New genetic mutations identified in the WNK1 gene, which especially alter the interaction of KS-WNK1 with the KLHL3/CUL3 ubiquitination complex, result in increased KS-WNK1, activation of SPAK and NCC, in addition to marked reduction of ROMK in DCT2 and principal cells (57). Patients with the reported WNK1 mutation develop hyperchloremic hyperkalemia, without arterial hypertension. The identification of these mutations is further evidence of the important role of KS-WNK1 in K^+^ homeostasis.

We can think of K^+^ homeostasis in the distal nephron as a set of scales that controls the reabsorption of Na^+^ between the DCT and the CNT/CCD. If plasma K^+^ levels are low, the reabsorption of Na^+^ must occur in the distal nephron mainly through the electroneutral transporter NCC. Conversely, if plasma K^+^ levels increase, the reabsorption of Na^+^ in the distal nephron must occur mainly through the electrogenic transporter ENaC, which makes the tubular lumen more negative and promotes K^+^ secretion. The plasma K^+^ concentration seems to be the “signaling element” for the allocation of Na^+^ reabsorption to one or other of these segments of the distal nephron.

## Maxi-K channel (BK) in high K^+^ diet

Maxi-K channel or big-K (BK) channel also contributes to K^+^ balance in high K^+^ diets. BK channels, with an alpha subunit that forms the channel and four different beta subunits (beta 1-4), expressed in principal cells (alpha1/beta1) and in intercalated cells (alpha1/beta4), where they are particularly abundant, are activated by increase in intracellular calcium and by higher tubular flow (58,59). High K^+^ diet induces a significant increase in distal nephron tubular flow due to loop inhibiting effect, increasing the BK channels activity and the K^+^ chemical gradient, both contributing to higher K^+^ secretion. The K^+^ uptake through the basolateral membrane of ICs seems to occur through the Na^+^-K^+^-2Cl^-^ cotransporter (NKCC1), since Na^+^/K^+^-ATPase is very low expressed in ICs. The BK channel activation in ICs, which normally bulge into the lumen, is relevant to decrease their volume and increase the luminal diameter of the tubule, which increases the fluid flow (60), contributing to the activation of both alpha1/beta1 and alpha1/beta4 BK.

## Basolateral K^+^ conductance and NCC activity

The central role of DCT in the fine daily regulation of K^+^ levels in the extracellular environment was further supported by the analysis of animals with removal of the K^+^ channel Kir4.1 in renal tissue (61). The Kir4.1/Kir5.1 heterotetramer is the K^+^ channel responsible for the potassium conductance in the basolateral membrane of DCT cells (12). Although this channel is also present in TAL, CNT, and CCD, there are other K^+^ channels in the basolateral membrane of these segments, so the significant depolarization of the basolateral membrane due to Kir4.1 deletion is observed only in DCT (62-64). The Kir4.1 K^+^ channel is fundamental for the functionality of the NCC cotransporter. Mice with a Kir4.1 deletion have manifestations similar to those observed in Gitelman syndrome (65). Kir4.1 deletion in the DCT cells has the same effect as increased plasma K^+^: the depolarization of the basolateral membrane results in higher intracellular Cl^-^ activity, which inhibits the WNK4/SPAK pathway signaling and reduces NCC activity. The opposite should be observed when the K^+^ conductance of the basolateral membrane is increased, since the membrane hyperpolarizes, as occurs when plasma K^+^ concentration is decreased.

## Regulation of DCT by the renin-angiotensin-aldosterone system (RAAS)

One very important mechanism for the renal handling of Na^+^ and K^+^ is the RAAS. The RAAS is activated in response to a reduction in the arterial pressure in thoracic and renal baroreceptors, and reduction of NaCl load to macula densa. These alterations cause an increase in renin production by the granular juxtaglomerular cells, the first step in the activation of the RAAS. Renin then proceeds through the cleavage of angiotensinogen produced in the liver into angiotensin I, which is converted into angiotensin II by the angiotensin I converting enzyme (ACE1). Angiotensin II (AngII) acts mainly by activation of the AngII receptor type I (AT1R), ubiquitously expressed, including the cells of the adrenal cortex, where it induces aldosterone (Aldo) secretion. The main consequence of activating the RAAS is the correction of hypovolemia, and to accomplish that, Aldo increases NaCl reabsorption in the aldosterone sensitive nephron (ASN). Aldo secretion can also be stimulated by high plasma concentrations of potassium, independent of AngII, which is decreased with K^+^ overload due to suppression of renin secretion (66). Therefore, three important hydro-electrolyte disturbances interfere differently with the RAAS: volume depletion increases AngII and Aldo, hyperkalemia increases Aldo and decreases AngII, and hypokalemia increases AngII and may decrease Aldo. The regulatory mechanisms dependent on AngII and Aldo must keep both Na^+^ and K^+^ homeostasis without conflict with each other. This fact has been known for decades as the aldosterone paradox, and fortunately the details of the complex signaling processes involved in these regulations have been progressively elucidated.

Together with CNT and CCD, DCT is referred to as the ASN due to the expression of 11beta-hydroxysteroid dehydrogenase type 2 (11beta-HSD2), which irreversibly converts cortisol into the inactive cortisone, making the selective activation of the mineralocorticoid receptor (MR) by Aldo possible. MR has an almost ubiquitous expression and is detected in all the distal nephrons, but 11beta-HSD2 is undetectable in DCT1; 11beta-HSD2 is expressed in DCT2, and its expression increases toward CNT and CCD (67); however, it is absent in intercalated cells (68). In DCT, specific Aldo effect is detected only in DCT2, where NCC and ENaC are coexpressed, and it was demonstrated that these proteins directly interact; this interaction is increased by Aldo, and inhibition of NCC by thiazide also inhibits ENaC in the complex (69,70).

The serum- and glucocorticoid-regulated kinase, *Sgk1,* is an early gene activated by Aldo, and one of the most important actions of SGK1 is to inhibit the ubiquitin ligase NEDD4-2 by inducing its phosphorylation. Non-phosphorylated NEDD4-2 promotes degradation of ENaC (71), NCC (72), and Kir4.1/Kir5.1. NEDD4-2 regulates Kir4.1/Kir5.1 expression/activity in the DCT by directly interacting with Kir5.1, the regulatory subunit of the K^+^ channel, resulting in ubiquitin-induced degradation of the K^+^ channel (73). In cells with activated SGK1 and consequent inhibition of NEDD4-2, the protein amount of NCC and Kir4.1/Kir5.1 channel might be increased by lower ubiquitin-mediated degradation; in addition, the hyperpolarization of DCT, due to increased K^+^ conductance of the basolateral membrane, also contributes to NCC activation by WNK4/SPAK pathway.

As highlighted above, WNK4 is a key regulator of transport in DCT, and both AngII and Aldo are very relevant for modulation of WNK4 activity. It was observed that AngII and Aldo, in adrenalectomized rats chronically treated with these hormones individually or in combination, increase phosphorylation of SPAK and NCC (74). The signaling pathways activated by AngII and Aldo can interfere directly with phosphorylation of WNK4 in different residues, but the electrolyte changes induced by Aldo, as hypokalemia, have also to be considered as important and independent modulators of WNK4 activity.

**Figure 6:**
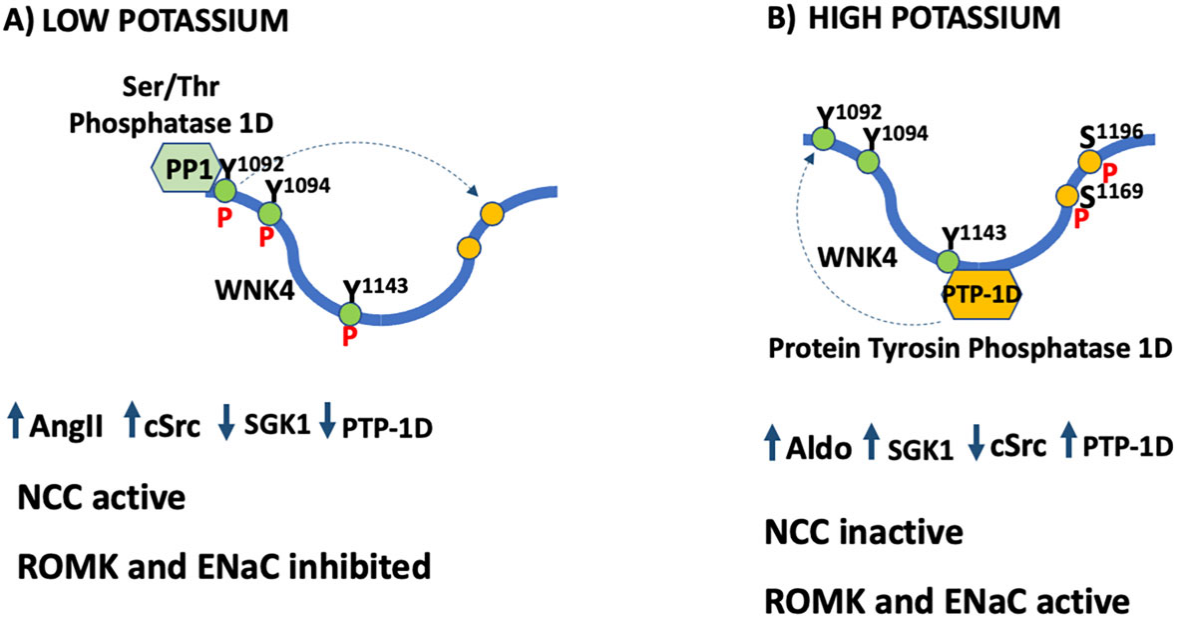
Angiotensin II and aldosterone modulate WNK4 phosphorylation in hypokalemia and hyperkalemia. **A**, Low K: WNK4 is phosphorylated by cSrc in Y 1092/1094/1143 and dephosphorylated in S 1969/1096 by the Ser/Thr phosphatase PP1, when angiotensin II (AngII) is high and aldosterone (Aldo) is low, and in this state, WNK4 activates Na-Cl cotransporter (NCC) and inhibits ROMK and ENaC. **B**, High K: WNK4 is phosphorylated by SGK1 in Ser 1069/1996 and dephosphorylated in Y 1092/1094/1143 by the protein tyrosine phosphatase PTP-1D, when AngII is low and Aldo is high, and in this state, WNK4 does not activate NCC, but activates ENaC and ROMK.

At least two relevant signaling pathways are activated by AngII binding to AT1R, Src family of tyrosine kinases (SFK) and PKC, and both phosphorylate WNK4. Aldo binding to MR, in turn, increases SGK1, a Ser/Thr kinase that also phosphorylates WNK4. Lin et al. (75), using HEK293T for transient expression of the proteins including WNK4, SGK1, cSrc, and ROMK channels, shed some light on regulation of WNK4 activity by cSrc and SGK1 (76) (Figure 6). They have shown that c-Src phosphorylates the tyrosines 1092, 1094, and 1143 in WNK4; both cScr and tyrosine phosphatase type 1D (PTP1D) interact with WNK4, regulating the Tyr phosphorylation level of WNK4. WNK4 phosphorylated on tyrosines, mainly Tyr^1092^, inhibits ROMK and ENaC by stimulating clathrin-mediated endocytosis, and activates SPAK, consequently activating NCC. In addition to ROMK inhibition by Tyr-phosphorylated WNK4, c-Src directly phosphorylates ROMK on Tyr^337^, increasing the channel endocytosis (77,78). SGK1 phosphorylates WNK4 on serine 1169 (79) and serine 1196 (80); SGK1 Ser^1169^ phosphorylation alleviates WNK4 inhibition of both channels, ENaC and ROMK, reverting the AngII effect, because pSer^1169^ facilitate the recruitment of the protein tyrosine phosphatase 1D (PTP-1D), which dephosphorylates tyrosine phosphorylated by cSrc. Lin et al. (75) speculated that pThy^1092^ promotes the recruitment of phosphatase PP1, which dephosphorylates the residues phosphorylated by SGK1. The results of these studies show that the modulatory effect of WNK4 on NCC, ENaC and ROMK can be tuned by AngII and Aldo, which modulate the level of WNK4 phosphorylation in different residues.

Increased PKC activity induced by AngII also activates WNK4 by phosphorylating KLHL3 and inhibiting WNK4 degradation induced by the KLHL3/CUL3 complex, and by phosphorylating the T-LOOP of the enzyme, a step necessary for its activation (81). Shibata et al. (82) have shown that PKC phosphorylates KLHL3 in the kelch domain, which precludes the interaction of the ubiquitination complex with WNK4, resulting in higher levels of WNK4 and activation of SPAK/NCC.

Volume depletion is a powerful stimulus of salt and water preservation, beginning by reducing filtration and by stimulating for Na^+^ reabsorption all the way through the nephron. Intercalated cells type B also contribute to the effort of Na^+^ preservation without K^+^ loss. Electroneutral Na^+^ reabsorption by B-ICs of CNT and CCD, through the combined action of NDCBE and pendrin, is activated by AngII and Aldo. Shibata et al. (83) have shown that in intercalated cells phosphorylation in Ser^843^ prevents Aldo or cortisol from binding to MR. In volume depletion, however, the higher AngII level reduces Ser^843^ phosphorylation by a mechanism that seems to involve WNK4, as suggested by the increased levels of MR-pSer^843^ in WNK4-KO mice. In volume depletion, the combined action of AngII and Aldo in B-ICs increases v-H^+^-ATPase and pendrin; additionally, NDCBE seems to be upregulated through AngII-dependent, and apparently MR-independent, pathways (83-85). In volume depletion, urinary Na-fractional excretion (FE-Na) is decreased, and K-fractional expression (FE-K) should not be changed, a goal achieved by predominant electroneutral Na^+^ reabsorption. B-ICs contribute to Na^+^ and K^+^ homeostasis in this condition by increasing NaCl electroneutral reabsorption.

Hyperkalemia inhibits electroneutral Na^+^ reabsorption in DCT and increases Na^+^ reabsorption by ENaC and K^+^ secretion by ROMK and maxi-K channel, in such a way that FE-Na is preserved and FE-K is increased. With a high K^+^ diet, WNK4, predominantly phosphorylated by SGK1 and inhibited by high [Cl^-^]_i_, has a low effect on activating NCC and does not inhibit ENaC and ROMK. Hypokalemia, on the other hand, increases NCC activity through WNK4-SPAK, at the same time that ENaC and ROMK are inhibited by WNK4. In this condition, it is expected that the FE-Na be preserved, at the same time that FE-K be decreased. WNK4 is centrally involved in this regulatory process.

In face of these three extremes of physiological conditions, hyperkalemia, hypokalemia, and volume contraction, we discuss the expected mobilization of regulatory mechanisms to preserve volume and potassium homeostasis. In pathological conditions, in which the normal negative feedback mechanisms are disrupted, such as primary or secondary hyperaldosteronism, it is less predictable how the combined levels of AngII, Aldo, and plasma K^+^ will affect the WNK4 activity and the activity of the transporters in the Aldo-sensitive nephron, and all these variables must be carefully analyzed to understand hydroelectrolytic and acid-base changes.

## NEDD4-2 in regulation of DCT

NEDD4-2 interferes with distal nephron ion transport in several ways. The E3 ubiquitin ligase NEDD4-2 is involved in degradation of ENaC, NCC, and the K^+^ channel Kir4.1/Kir5.1, and the activity of these transporters is altered by NEDD4-2 in conditions such as low salt diet (volume depletion) and high potassium diet, in which aldosterone increases SGK1, the kinase involved in phosphorylation and inactivation of NEDD4-2. Wu et al. (73) observed that in ks-*Nedd4-2* KO mice there is overexpression of Kir4.1/Kir5.1, which increases the K^+^ permeability of the basolateral membrane of DCT cells, resulting in hyperpolarization and efflux of intracellular Cl^-^; there is activation of the WNK4/SPAK pathway, with increases in total and phosphorylated NCC (86). In ks-*Nedd4-2* KO mice, neither high K^+^ decreased nor low K^+^ increased basolateral Kir4.1/Kir5.1 activity (61,87) as observed in control mice. Deletion of *Nedd4-2* induces an increase in epithelial sodium channel α-subunit and abolishes the effect of high salt on decreasing expression and activity of ENaC, Kir4.1, and NCC, resulting in higher K^+^ excretion and hypokalemia under salt load (88). Kir4.1/Kir5.1 deletion results in depolarization of the basolateral membrane of DCT cells, decreases NCC activity by inhibiting the WNK4/SPAK pathway, increases the Na^+^ load to principal cells, and induces hypokalemia (12). The double *Nedd4-2/Kir4.1* KO mice, however, did not develop hypokalemia, as did *Kir4.1/Kir5.1* KO mice, probably because the deletion of *Nedd4-2* resulted in less NCC degradation and preservation of the electroneutral Na^+^ reabsorption in DCT (73) and lower Na^+^ load into principal cells.

**Figure 7:**
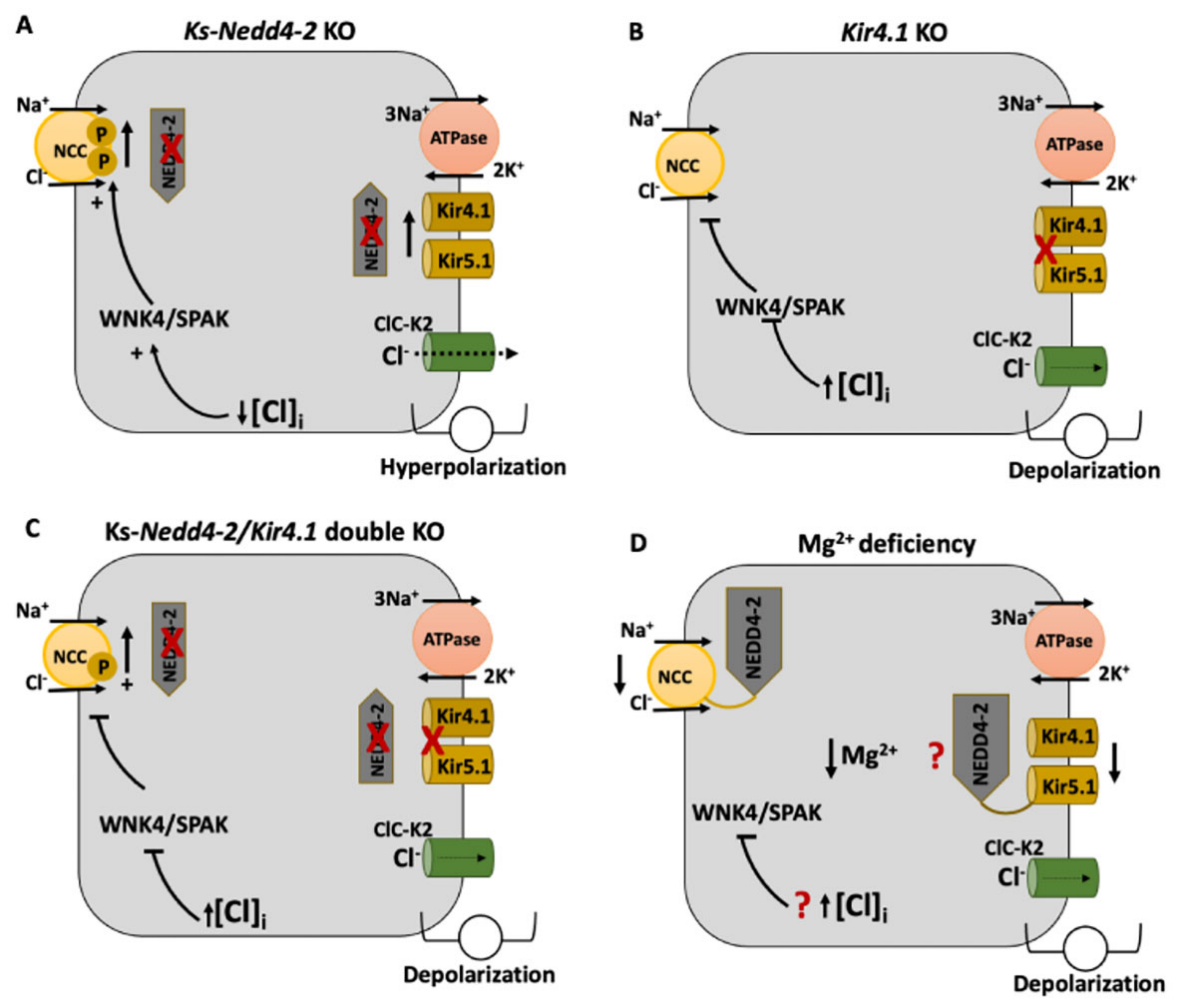
The ubiquitin ligase NEDD4-2 modulates the transport of Na^+^ and K^+^ in distal convoluted tubules (DCT). **A**, ks-Nedd4-2 KO mice show increase in total and phosphorylated amount of Na-Cl cotransporter (NCC) at the apical membrane and in the activity of the Kir4.1/Kir5.1 K^+^ channel at the basolateral membrane of DCT cells, which results in hyperpolarization of the basolateral membrane, reduction in the intracellular Cl^-^ activity, and activation of WNK4/SPAK. **B**, ks-Kir4.1 KO mice show high intracellular Cl^-^ and low activity of WNK4/SPAK, which results in low amount of total and phosphorylated NCC. **C**, ks-Nedd4-2/Kir4.1 double KO mice show up-regulation of NCC compared to ks-Kir4.1 KO mice, which suggests that the NCC degradation mediated by NEDD4-2 was abolished. **D**, Magnesium deficiency is associated with downregulation of NCC by a mechanism dependent on NEDD4-2, suggesting that NEDD4-2 is activated by magnesium depletion.

It is a frequent observation that Mg^2+^ deficiency exacerbates the effect of hypokalemia; hypokalemia with concomitant Mg^2+^ deficiency is often refractory to K^+^ supplement (89). Mg^2+^ deficiency downregulates total NCC abundance and it does not directly involve WNK/SPAK pathway, but involves NEDD4-2, since in *ks-Nedd4-2* KO mice, NCC abundance was not lowered with Mg^2+^ restriction (90), as observed when NEDD4-2 is normally expressed. Mg^2+^ deficiency, by a still unknown mechanism, seems to activate NEDD4-2, which in turn mediates degradation of NCC and blunts the expected effect of hypokalemia on activation of NCC. Figure 7 summarizes the effects of *Ncc*-KO, *Kir4.1*-KO, Ncc/*Kir4.1* double KO, and Mg^2+^ deficiency on NEDD4-2 activity and on NCC. It was not investigated whether Mg^2+^ deficiency also induces a decrease in Kir4.1/Kir5.1 protein abundance and an inhibition of the WNK4/SPAK signaling pathway.

## Conclusions

The main goal of this review was to highlight the central role of the DCT in the control of K^+^ homeostasis and how the electrogenic or electroneutral Na^+^ reabsorption in distal nephron is modulated by complex mechanisms in which new players, such as WNK4, pendrin/NDCBE, MR in B-IC, NEDD4-2, are involved, and certainly, many others are yet to be investigated. In conclusion, we list the most relevant observations of the last decades, which contribute to making an understandable picture of the role of DCT, associated with CNT and CCD, in Na^+^ and K^+^ homeostasis.

1. In addition to NCC, DCT cells approaching CNT express the epithelial sodium channel (ENaC) in interaction with NCC, which contributes for K^+^ secretion. The ICs can be detected among the DCT2 cells and may be a pathway for chloride reabsorption.

2. The allocation of Na^+^ reabsorption to the DCT cells or to the principal cells of CNT and CCD is essential to modulate K^+^ secretion, and it is intimately determined by the plasma K^+^ levels.

3. The central role of the kinase WNK4 in modulation of NCC activity was discovered when mutations on *Wnk* genes were responsible for around 20% of the clinical manifestations of pseudohypoaldosteronism type II (PHAII), characterized by hyperkalemia and hypertension, due to hyperactivity of NCC. Mutations on *Klhl3*/*Cul3* genes, which encode ubiquitination complex interacting with WNKs, are responsible for almost all other genetic mutations related to PHAII, and the final manifestations are due to increased WNK kinases (WNK1 and WNK4) activity.

4. The NCC activation by WNK4 depends on activation of the kinases SPAK and OSR1, which eventually phosphorylate and activate NCC. Depending on its phosphorylation pattern, WNK4 can activate NCC through SPAK/OSRI or inhibit NCC by diverting it to lysosomal degradation. WNK4 modulates other transporters in distal nephrons, including ROMK and ENaC, decreasing their activity by stimulating lysosomal degradation, or increasing their activity when degradation is not stimulated.

5. WNK4 has a Cl^-^ interaction domain and functions as an intracellular chloride sensor. Chloride binding inhibits WNK4 autophosphorylation, which is necessary for its activity. The basolateral membrane electrical potential determines the intracellular Cl^-^ concentration: hyperpolarization of this membrane, as observed when plasma K^+^ concentration is low or when the K^+^ conductance of the basolateral membrane is increased, induces Cl^-^ efflux and WNK4 activation. The opposite is observed when plasma K^+^ concentration is high or when basolateral K^+^ channel (Kir4.1/Kir4.5) is downregulated or deleted. Experimental evidence suggests that daily fluctuations of plasma K^+^ level are a relevant mechanism of NCC activity modulation by WNK4 and control of K^+^ secretion by principal cells, which is highly dependent on Na^+^ loading to CNT and CCD.

6. The RAAS, central in regulation of the transport in the aldosterone sensitive nephron, is involved in the WNK4 activity modulation. Both, ANGII and Aldo activate a set of kinases, which modulates WNK4 activity in specific ways. AngII activates cSrc and PKC kinases and Aldo activates SGK1.

7. WNK4 phosphorylated by cSrc in tyrosines, mainly Thyr^1992^, activates SPAK, increasing pNCC, and inhibits ROMK and ENaC. In addition, PKC activation results in KHLH3 phosphorylation and inhibition of the WNK4 ubiquitination complex. On the other hand, in the absence of cSrc activation, SGK1 increases the WNK4 Ser^1992/1996^ phosphorylation, which allows the recruitment of a Tyr-phosphatase, which removes Thyr^1992^ phosphorylation, precludes SPAK/NCC activation and liberates the activity of ENaC and ROMK; in addition, SGK1 activates ENaC by several mechanisms. By these mechanisms, high AngII and low Aldo, which can be observed in hypokalemic conditions, favors Na^+^ and Cl^-^ reabsorption without K^+^ loss; and low AngII and high Aldo, which can be observed in hyperkalemic conditions, favors K^+^ secretion without change in Na^+^ excretion.

8. In extracellular volume contraction, a condition in which both AngII and Aldo are increased, the whole nephron is recruited to preserve salt and water, considerably reducing the Na^+^ loading to the distal nephron, which reabsorbs practically the total amount of Na^+^ that reaches CNT and CCD. Although in ICs the MR receptor is phosphorylated in the ligand binding domain, which blunts Aldo or cortisol binding to the receptor, the increase in AngII results in dephosphorylation and activation of MR in these cells. Since ICs do not express 11-beta HSD2, it is possible that cortisol activates the receptor. The increased electroneutral Na^+^ reabsorption by the combined activity of pendrin and NDCBE B-IC has been shown to be important to prevent severe hypotension in volume contraction, as observed in the double KO *Ncc/pendrin*.

9. The intense diuretic effect of the double KO *Ncc/pendrin*, also due of the ENaC and APQ2 inhibition associated to pendrin deletion and not associated to hypokalemia due to the considerable inhibition of ENaC, suggests that the combination of thiazide diuretics with a pendrin inhibitor could be very useful for patients with severe extracellular volume increase.

10. NEDD4-2, the E3-ubiquitin inhibited by SGK1-induced phosphorylation, is involved in ubiquitination and proteasomal degradation, not only of ENaC, but also of NCC and K4.1/Kir5.1 K^+^ channel, which is related to persistent hypokalemia observed in patients receiving thiazide diuretics, as reported by the absence of this important side effect of thiazide in *Nedd4-2*-KO animals. The Mg^2+^ intracellular depletion in the DCT cells induced by thiazides activates NEDD4-2, and NEDD4-2 induces NCC degradation, which blunts the expected hypokalemic activation of NCC.
